# New faecal tests for colorectal cancer screening: is tumour pyruvate kinase M2 one of the options?

**DOI:** 10.1038/sj.bjc.6604039

**Published:** 2007-10-23

**Authors:** Y M Shastri, J M Stein

**Affiliations:** 1 Department of Medicine I-ZAFES, Division of Gastroenterology and Clinical Nutrition, Johann Wolfgang Goethe-University Hospital, Frankfurt am Main, Germany

**Sir**,

We read with great interest the article by Haug *et al* (2007) and must complement them for a nicely written work. We have certain comments to make about their study.

Colorectal adenomas, the precursors of almost all of the sporadic colorectal cancer (CRC) could not be included in their study. The ability of various CRC screening programme (eg, colonoscopy) in reducing CRC mortality by diagnosing and removing these premalignant lesions cannot be overemphasised. It is agreed that the false positives due to these adenomas in the control group would have contributed to marginal increase in the specificity of faecal pyruvate kinase type M2 (M2-PK) in their study (Haug *et al*, 2007), as rightly mentioned by the authors.

But what about the adenomas that could have been missed out by performing M2-PK assay in the controls? The prevalence of any adenomatous lesion in their control group would have been somewhere around 30% and roughly 8% would have been advanced adenomas (Anderson *et al*, 2003), usually described as villous adenomas, tubular adenomas >10 mm, high-grade dysplasia, malignant polyp or >3 adenomas (ie, around 73 out of these 917 control group patients (Haug *et al*, 2007)). The previous studies have reported sensitivity of M2-PK to be just 30% in diagnosing advanced adenomas (Shastri *et al*, 2006) and 40% by Koss *et al* (2005) (only as an abstract). Thus, assuming that a sensitivity of around 35% for diagnosing these advanced adenomas from the above two studies would be applied to the population studied, then there would be a marked decrease in the resulting sensitivity of M2-PK for diagnosing colorectal neoplasia (CRN), that is CRC with advanced adenomas. Thus, the estimated sensitivity of M2-PK in diagnosing CRN would decrease to 48% (26+45/73+74) as against 67% (45/74) reported in this study (Haug *et al*, 2007). This would be highly unacceptable for any CRC screening test and especially for M2-PK with such a poor specificity of around 80%, which is much lower than that of any of the faecal screening tests for CRC, in fact even lower than that of Guaiac-based faecal occult blood test (FOBTs).

One of the limitations of their study was a retrospective study design with historical controls; these subjects did not undergo either any of the more accurate faecal screening test, for example, IFOBT (immunological FOBT) or a colonoscopy for confirming the results of M2-PK in detecting CRC. Thus, if the prevalence of CRC is taken to be 1% in the historical controls, there lies a possibility of missing out significant number of CRC in the control group considering such a low performance characteristics which is highly unacceptable.

We agree with the authors that the poor performance characteristics (especially low specificity and poor PPV) of faecal tumour M2-pyruvate kinase (M2-PK) as has also been reported in previous prospective study (Shastri and Stein, 2007) does not warrant its use as a marker for CRC screening. This is particularly relevant when other better options are available for stool testing for CRC namely IFOBT (Allison *et al*, 1996; Morikawa *et al*, 2005) and should have been discussed by the authors. These ELISA-based IFOBTs (Allison *et al*, 1996) or automatically analysable IFOBT (Morikawa *et al*, 2005) have demonstrated sensitivity of around 60% and specificity of about 90%. Recently various ‘office based or bedside’ simple, convenient and cheap strip-based IFOBT have been validated for CRC screening with performance characteristics similar to that of an ELISA-based IFOBT (Hoepffner *et al*, 2006; Smith *et al*, 2006). In fact our group compared an office-based IFOBT with that of M2-PK to show that M2-PK and IFOBT had a sensitivity of 80.4 *vs* 72.3% for diagnosing CRN, whereas specificity for these tests were 76.3 *vs* 94.7%, respectively (Shastri *et al*, 2007). All the above information further demonstrates it emphatically that M2-PK cannot be recommended as a marker for CRC screening.

Considering above factors it would be really catastrophic to recommend M2-PK as a biomarker for screening patients with CRC when much better options for the same are available.
